# BamA as a next-generation antibacterial target: mechanistic insights, inhibitor landscape, and rational drug design strategies

**DOI:** 10.1039/d6ra06788c

**Published:** 2026-10-13

**Authors:** Ahmed A. Al-Karmalawy, Arwa Omar Al Khatib, Samia S. Hawas

**Affiliations:** a Department of Pharmaceutical Chemistry, Faculty of Pharmacy, Horus University-Egypt New Damietta 34518 Egypt akarmalawy@horus.edu.eg; b Faculty of Pharmacy, Al-Ahliyya Amman University Amman Jordan

## Abstract

The rapid emergence of antimicrobial resistance (AMR) among Gram-negative pathogens has intensified the need for novel therapeutic strategies targeting previously underexploited bacterial systems. The β-barrel assembly machinery (BAM) complex, and particularly its central component BamA, has emerged as a highly promising antibacterial target due to its essential role in outer membrane protein (OMP) biogenesis and its accessibility at the bacterial surface. This review provides a comprehensive analysis of BamA structure, function, and mechanism, with emphasis on the dynamic lateral gate and β-augmentation processes that drive OMP folding and insertion. Recent advances in structural biology have enabled detailed characterization of BamA conformational states, offering critical insights into its druggability. We further present an integrated overview of the current BamA inhibitor landscape, including macrocyclic peptides, peptidomimetics, and small molecules, highlighting key structure–activity relationships (SAR) and binding modes. In addition, mechanisms of resistance, challenges associated with outer membrane (OM) permeability, and pharmacokinetic limitations are critically discussed. Particular attention is given to emerging design strategies that integrate conformational targeting, β-strand mimicry, and permeability optimization. Collectively, this review underscores the potential of BamA as a next-generation antibacterial target and outlines future directions for the development of effective therapeutics against multidrug-resistant Gram-negative bacteria.

## Abstract 2

BamA as a next-generation antibacterial target. Graphical abstract generated using Google Gemini (Nano Banana).

## Graphical abstract

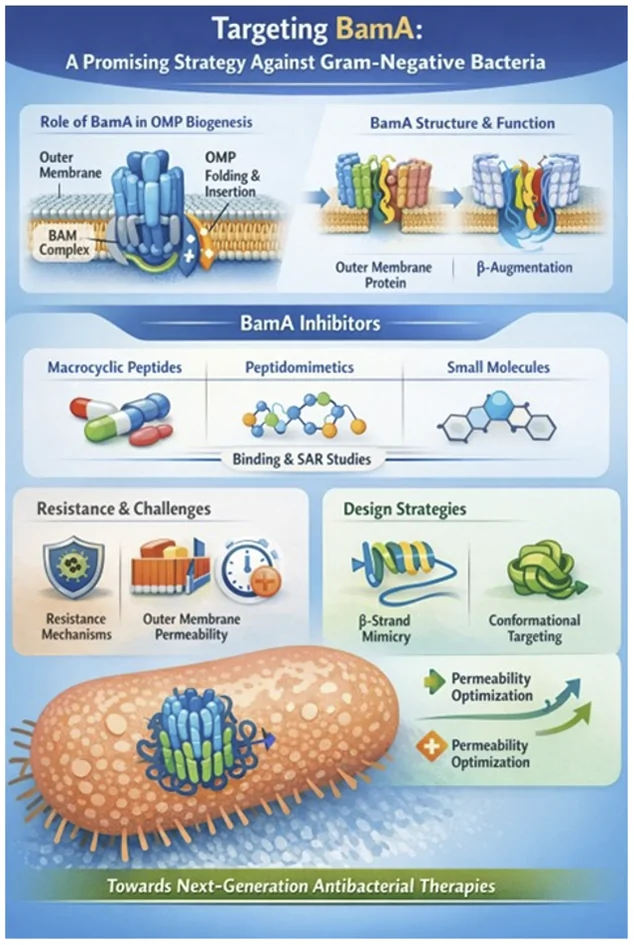

## 1 Introduction

Antimicrobial resistance (AMR) has escalated into one of the most urgent and complex global health challenges of the modern era.^1–4^ Recent assessments indicate that drug-resistant infections are exerting an increasingly severe toll on global health systems, with their impact expanding each year and placing growing pressure on medical infrastructure and patient care worldwide.^5–8^ This crisis is particularly severe for Gram-negative bacterial pathogens, which dominate the World Health Organization's critical-priority list due to their escalating resistance to nearly all major antibiotic classes—including carbapenems, polymyxins, and even some of the newest β-lactam/β-lactamase inhibitor combinations.^9^

A defining feature of Gram-negative bacteria is their double-membrane cell envelope, consisting of an inner membrane and a highly impermeable outer membrane (OM).^10,11^ The OM acts as a robust chemical and physical shield, preventing many antibiotics from reaching intracellular targets.^12^ Its asymmetric lipid bilayer, enriched with lipopolysaccharide (LPS), creates an exceptionally restrictive permeability barrier that excludes hydrophobic molecules and limits the diffusion of many hydrophilic agents through selective porins.^13,14^ As a result, Gram-negative species routinely evade the action of antibiotics that remain effective against Gram-positive organisms.^15–18^ This intrinsic barrier, combined with their ability to acquire mobile genetic elements such as plasmids, integrons, and transposons, makes Gram-negative pathogens highly proficient at evolving multidrug resistance, broadening their ecological persistence, and complicating therapeutic outcomes.^19,20^

Within this protective OM lies a critical vulnerability: the β-barrel assembly machinery (BAM) complex, an essential multiprotein system responsible for the folding, insertion, and maturation of outer membrane proteins (OMPs).^21,22^ OMPs play indispensable roles in maintaining membrane integrity, facilitating nutrient uptake, mediating host–pathogen interactions, and contributing to virulence.^23,24^ Their biogenesis follows a highly coordinated pathway in which precursor proteins are synthesized in the cytoplasm, translocated across the inner membrane *via* the SecYEG translocon, and transported through the periplasm before being delivered to the BAM complex for final membrane integration.^25,26^ Disruption of this pathway leads to global defects in OM biogenesis, loss of membrane integrity, and ultimately bacterial death, highlighting the essential nature of the BAM system.^27^

Among the five BAM subunits (BamA–E), BamA serves as the central and indispensable component. It belongs to the OMP85 protein family and is highly conserved across virtually all Gram-negative bacteria.^28,29^ Structurally, BamA consists of an N-terminal periplasmic domain containing multiple polypeptide transport-associated (POTRA) motifs and a C-terminal 16-stranded β-barrel embedded in the OM.^30,31^ Functionally, BamA orchestrates OMP assembly through dynamic conformational transitions, including the regulated opening of its lateral gate, which enables β-strand templating and insertion into the membrane.^32,33^ These conformational dynamics generate transient and potentially druggable binding sites, offering unique opportunities for rational inhibitor design from a medicinal chemistry perspective.

Importantly, BamA fulfills several key criteria of an ideal antibacterial target: it is essential for bacterial viability, highly conserved, and partially surface-exposed, allowing direct accessibility to extracellular inhibitors.^34,35^ Targeting BamA and the BAM complex therefore represents a promising strategy to overcome the intrinsic permeability barrier of Gram-negative bacteria and to disrupt OM biogenesis at its core. This concept aligns with emerging therapeutic approaches aimed at “breaking the OM barrier” by directly interfering with OM assembly pathways.^36,37^

Recent advances have validated this strategy, with the discovery of several classes of BamA inhibitors, including natural products such as darobactins, as well as synthetic macrocyclic peptides and peptidomimetics like xenorhabdins and xenorceptides.^38–41^ These compounds demonstrate that BamA is not only druggable but also capable of yielding potent antibacterial agents with novel mechanisms of action. Such inhibitors often exploit the dynamic structural features of BamA, stabilizing inactive conformations or blocking substrate interaction sites, thereby preventing proper OMP folding and insertion (Fig. 1).

**Fig. 1:**
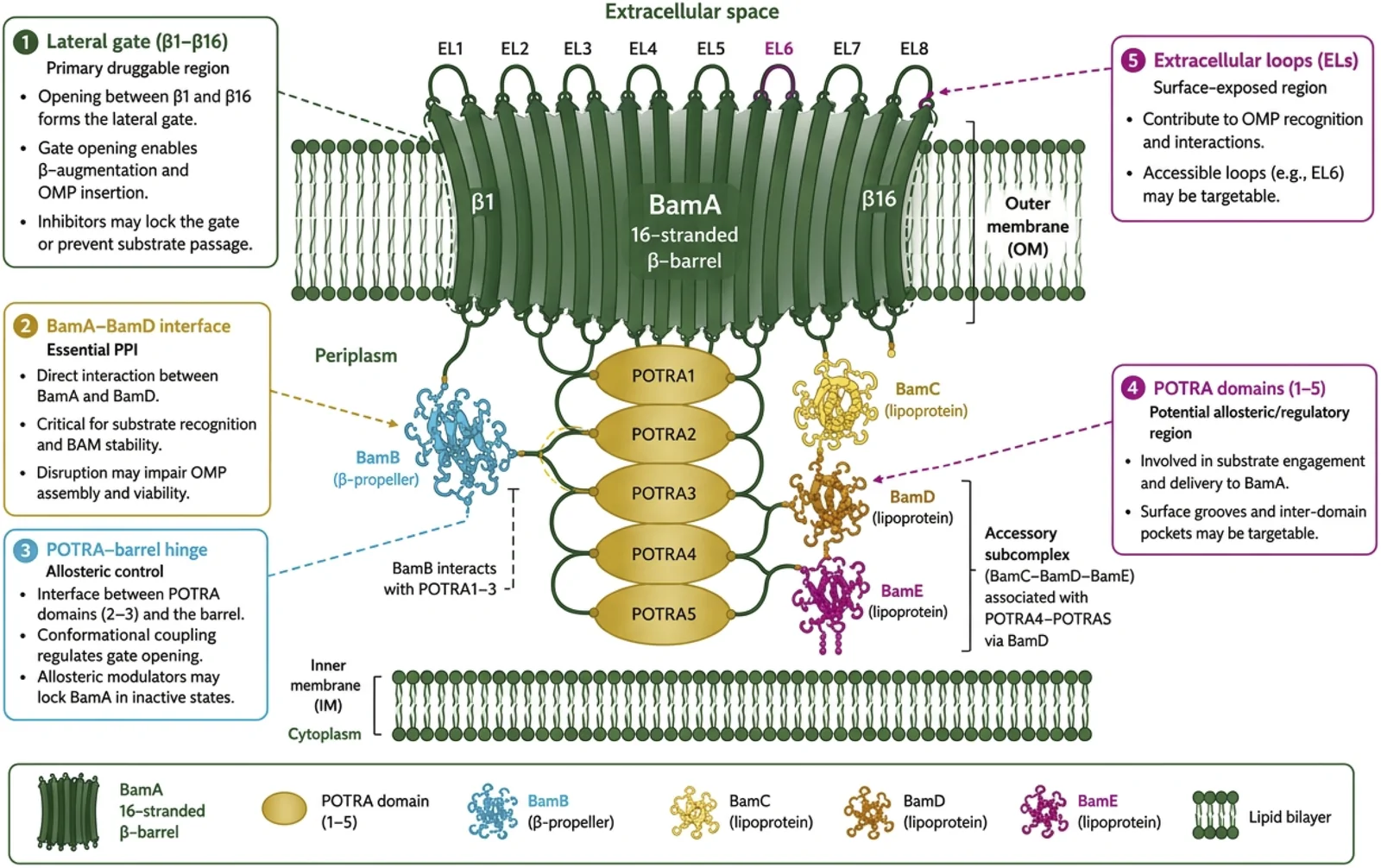
Architecture of BamA and the BAM complex in Gram-negative bacteria and major pharmacologically relevant regions. BamA is an outer-membrane β-barrel protein containing five N-terminal periplasmic POTRA domains and a C-terminal 16-stranded β-barrel. The accessory lipoproteins BamB–E associate with BamA on the periplasmic side of the outer membrane. Major pharmacologically relevant regions include the lateral gate formed by β1 and β16, extracellular loops accessible to surface-targeting agents, the POTRA domains involved in substrate recognition and delivery, and protein–protein interaction interfaces within the BAM complex (generated with NanoBanana).

Collectively, these findings position BamA at the forefront of next-generation antibacterial drug discovery.^42–45^ A comprehensive understanding of its structure, mechanism, and inhibitor landscape is therefore essential for guiding the rational design of innovative therapeutics aimed at combating multidrug-resistant Gram-negative infections and addressing the escalating global AMR crisis.

Unlike previous reviews that primarily focus on structural or biochemical aspects of the BAM, this review provides an integrated medicinal chemistry perspective that connects BamA structural dynamics, β-strand-mediated substrate recognition, and inhibitor design principles. Particular emphasis is placed on the role of conformational flexibility and lateral gate dynamics as central determinants of druggability, highlighting how these features can be exploited for rational inhibitor development. In addition, this work systematically correlates structure–activity relationship (SAR) insights from diverse inhibitor classes with their underlying mechanistic modes of action. By combining structural biology, resistance evolution, and pharmacokinetic constraints into a unified framework, this review offers a comprehensive roadmap for the design of next-generation BamA-targeting antibiotics.

## 2 Structural biology of the BAM complex

### 2.1 Composition and architecture

The BAM complex represents the central system responsible for the biogenesis of OMPs in Gram-negative bacteria. In *Escherichia coli*, this complex is composed of five distinct subunits—BamA, BamB, BamC, BamD, and BamE—which assemble into a coordinated and dynamic molecular machine that ensures the proper folding and insertion of β-barrel proteins into the OM.^46,47^

At the core of the BAM complex lies BamA, an essential and highly conserved protein that defines both the structural and functional framework of the system. BamA consists of a transmembrane 16-stranded β-barrel domain embedded within the OM, coupled with a large periplasmic region composed of five POTRA domains (POTRA1–5). These POTRA domains form an extended and flexible scaffold that mediates interactions with substrate proteins as well as with the accessory BAM components. Structural and biophysical studies have demonstrated that this region exhibits significant conformational plasticity, which is thought to be critical for accommodating unfolded OMP substrates and coordinating their stepwise assembly.^48,49^

BamD, a tetratricopeptide repeat (TPR)-containing lipoprotein, forms—together with BamA—the essential functional core of the complex. It directly interacts with the POTRA5 domain of BamA, establishing a stable and indispensable interface required for bacterial viability. This BamA–BamD interaction acts as a central regulatory hub, coordinating substrate recognition and communication between periplasmic and membrane-embedded regions. Disruption of this interface leads to severe defects in OMP assembly and cell death, underscoring its biological and pharmacological importance.^47,50^

The remaining subunits—BamB, BamC, and BamE—are non-essential but play critical modulatory roles. BamB, a β-propeller protein, interacts primarily with POTRA2–3 and enhances the efficiency and fidelity of OMP folding.^46,51^ BamC associates indirectly with BamA *via* BamD and contributes to conformational rearrangements within the complex, while BamE stabilizes the BamA–BamD interaction and maintains overall complex integrity.^52–54^

High-resolution structural studies (cryo-EM and X-ray crystallography) reveal a characteristic “hat-shaped” architecture, where the BamA β-barrel forms the membrane-embedded crown and the POTRA domains with associated lipoproteins form a brim-like periplasmic assembly. Within this arrangement, BamB is positioned proximal to the mid-POTRA domains (notably POTRA3), whereas BamC, BamD, and BamE form a tightly associated subcomplex that localizes near POTRA4–5, establishing a coordinated interaction network that supports efficient substrate handling and insertion.^52,55^

From a medicinal chemistry perspective, this architecture exposes multiple pharmacologically addressable interfaces, positioning the BAM complex as a highly attractive antibacterial target.

### 2.2 BamA structural dynamics

BamA is not a static scaffold but a highly dynamic membrane insertase whose conformational flexibility is central to its function. A defining structural feature is the lateral gate formed between β-strands β1 and β16, which acts as a dynamic seam capable of transitioning between closed and open conformations.^56–59^

In the closed state, β1 and β16 maintain partial hydrogen bonding, though weaker than the rest of the barrel, predisposing the seam to opening. In the open state, these strands separate, creating a lateral opening toward the lipid bilayer that enables substrate insertion.^56–58^ Structural and biophysical studies—including cryo-EM, NMR, ESR, and molecular dynamics (MD) simulations—consistently demonstrate that BamA cycles between these states during OMP biogenesis.^57,60^

Mechanistically, lateral gate opening enables β-augmentation, whereby nascent OMP β-strands transiently pair with the β1 strand of BamA to form hybrid barrel intermediates.^61–64^ This supports a sequential folding mechanism in which OMPs are progressively assembled and inserted into the membrane.

In parallel, the POTRA domains—particularly POTRA5—undergo coordinated conformational changes that are allosterically coupled to lateral gate dynamics. Depending on its position, POTRA5 can either occlude or expose the barrel lumen, thereby regulating substrate access and synchronizing periplasmic substrate delivery with membrane insertion.^56,57,65^

BamA also actively remodels the surrounding lipid environment. Cryo-EM studies in nanodiscs and MD simulations reveal localized membrane thinning near the lateral gate, which reduces the energetic barrier for OMP insertion.^66^ This highlights that BamA functions not only as a scaffold but also as a catalyst of membrane remodeling.

### 2.3 Coupling of POTRA domains and the lateral gate

The POTRA domains play a central role in substrate recognition, recruitment, and delivery. These domains interact with unfolded OMPs and periplasmic chaperones such as SurA, forming a dynamic and adaptable binding platform.^67^ Experimental evidence (*e.g.*, HDX-MS and neutron reflectometry) shows that substrate binding induces elongation and outward displacement of the POTRA domains, positioning substrates for transfer to the β-barrel domain.^31^

Building on the conformational dynamics described in Section 2.2, a key mechanistic feature is the allosteric coupling between POTRA domain movements and lateral gate transitions.^56,65^ Disruption of this coupling—such as by rigidifying the POTRA–barrel hinge—severely impairs BamA function, confirming the necessity of coordinated conformational flexibility.^56^

During OMP insertion, the lateral gate serves as the primary entry site. In the open state, substrate β-strands engage *via* their C-terminal β-signal, initiating β-augmentation and folding. In the closed or inward-open state, the barrel lumen becomes accessible, potentially representing a reset or substrate-engaged intermediate.^63,68^

The dynamics of this system are further modulated by accessory proteins (especially BamD), species-specific structural variations, and chaperone interactions, all of which fine-tune the conformational landscape of BamA.^65^

### 2.4 Druggable features and medicinal chemistry implications

The structural and dynamic complexity of the BAM complex translates directly into multiple druggable hotspots, making it a prime target for antibacterial drug design.

#### 2.4.1 Key pharmacologically addressable regions

From a medicinal chemistry perspective, several regions of BamA may provide opportunities for pharmacological intervention. The lateral gate formed by β1 and β16 represents a central mechanistic hotspot because of its role in substrate interaction and OMP insertion.^56–59^ The flexible POTRA domains may provide sites for allosteric modulation of substrate recruitment and conformational coupling, whereas the BamA–BamD interface represents a potentially valuable protein–protein interaction target.^54,57,63,69,70^ The POTRA–barrel hinge may offer an additional allosteric control point for interfering with conformational transitions.^30^ In addition, the extracellular loops of BamA are accessible from the bacterial surface and may therefore be particularly suitable for peptide- or biologic-based approaches.^71^ These structural and dynamic features form the mechanistic foundation for inhibitor design, which will be referenced throughout subsequent sections.

#### 2.4.2 Interfaces enable diverse targeting strategies

These structural features also support several complementary approaches to BamA inhibition. Direct interference with the lateral gate could prevent substrate engagement, whereas targeting POTRA dynamics or the hinge region may achieve inhibition through allosteric modulation.^72,73^ The BamA–BamD interface provides an alternative opportunity to disrupt protein–protein interactions, while substrate-mimetic strategies may interfere with β-signal recognition and β-strand engagement.^73,74^

Importantly, resistance mutations frequently localize to the lateral gate region, further validating its functional and pharmacological relevance.^75^ Licochalcone A has also been reported to interfere with OM homeostasis and has been proposed to affect the BamA/BAM pathway; however, direct structural or biochemical evidence confirming specific binding to BamA remains insufficient. It is therefore considered a phenotypic BamA-associated compound rather than a structurally validated direct BamA inhibitor.^76^

These structural and dynamic features form the mechanistic foundation for inhibitor design, which will be referenced throughout subsequent sections.

## 3 Mechanism of BAM and medicinal chemistry implications

### 3.1 OMP substrate recognition, delivery, and early folding events

The biogenesis of OMPs in Gram-negative bacteria follows a highly coordinated, multi-step pathway that ensures efficient delivery of unfolded substrates from the cytoplasm to the OM. This process involves sequential handoff between membrane translocons, periplasmic chaperones, and the BAM, with BamA acting as the central hub for substrate recognition and folding initiation (Fig. 2).

**Fig. 2:**
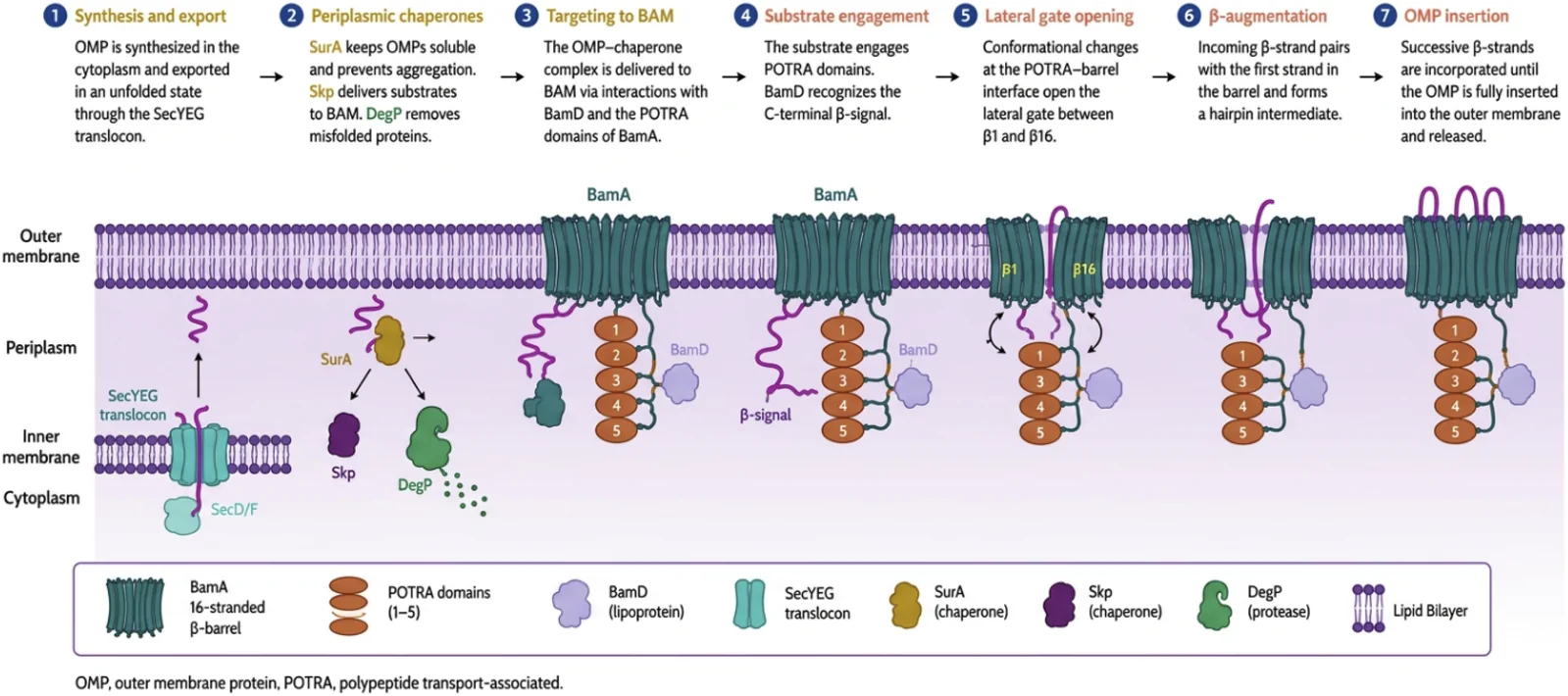
Mechanism of β-barrel outer membrane protein (OMP) assembly and pharmacologically relevant intervention points. OMP precursors are translocated across the inner membrane through the SecYEG pathway and maintained in an unfolded state by periplasmic chaperones before delivery to the BAM complex. BamA subsequently mediates substrate recognition, β-strand engagement, folding, and insertion into the outer membrane through coordinated conformational changes involving the POTRA domains and lateral gate. Potential therapeutic strategies include interference with substrate recognition, lateral-gate dynamics, conformational transitions, and protein–protein interactions within the BAM complex (generated with NanoBanana).

OMP precursors are synthesized in the cytoplasm with N-terminal signal peptides that target them to the SecYEG translocon for export across the inner membrane.^77–81^ During translocation, substrates remain in an unfolded conformation, which is necessary for passage through the Sec channel but renders them highly prone to aggregation in the periplasmic environment.^79^ To mitigate this risk, unfolded OMPs are rapidly captured by periplasmic chaperones.

Among these, SurA functions as the primary trafficking chaperone, directly interacting with both unfolded OMPs and BamA.^82,83^ Auxiliary chaperones such as Skp and DegP provide complementary roles, acting as holdases or proteases under stress conditions to stabilize or eliminate misfolded proteins.^79,80^ Notably, SurA can form transient ternary complexes with BamA and substrate OMPs, facilitating efficient substrate delivery and early folding coordination.^33^ In addition, growing evidence supports the existence of trans-envelope supercomplexes linking SecYEG, SecDF, YidC, SurA, and BAM, enabling direct substrate handoff and minimizing exposure to the crowded periplasm.^84–86^

Both sequence and structural determinants govern substrate recognition at the BAM complex. A critical feature is the C-terminal β-signal, which is selectively recognized by BamD and serves as a quality control checkpoint to ensure proper substrate targeting.^77,87,88^ Concurrently, BamA initiates folding through β-augmentation, whereby its N-terminal β-strand forms hydrogen bonds with the incoming substrate, nucleating β-barrel formation.^77,89^

The POTRA domains of BamA act as dynamic periplasmic scaffolds that guide substrates toward the membrane-embedded β-barrel domain. These domains interact with unfolded β-strand edges and exhibit intrinsic flexibility—particularly at the POTRA2–POTRA3 interface—allowing accommodation of structurally diverse substrates.^90,91^ Upon substrate engagement, POTRA domains undergo conformational rearrangements that position the substrate for delivery to the lateral gate.^92^ Restricting this flexibility through mutational constraints significantly impairs OMP assembly, confirming their active mechanistic role.^91,93^

From a medicinal chemistry perspective, this early stage of OMP biogenesis presents multiple opportunities for intervention. Disrupting SurA–BamA interactions, blocking β-signal recognition by BamD, or restricting POTRA domain flexibility can interfere with substrate delivery and folding initiation. Additionally, mimicking β-strand edges or backbone hydrogen bonding patterns represents a promising strategy to competitively inhibit substrate engagement.

### 3.2 Energy-independent insertion and conformational dynamics of BamA

A defining characteristic of the BAM complex is its ability to catalyze OMP insertion without external energy sources such as ATP hydrolysis or proton motive force. Instead, BAM operates through intrinsic conformational dynamics and thermodynamic driving forces, distinguishing it from most membrane protein insertion systems.^68,94^

Extensive studies confirm that OMP folding and insertion occur in an energy-independent manner, raising the question of how BAM overcomes the energetic barrier of membrane integration. Current models attribute this capability to the coordinated interplay between BamA structural flexibility, lateral gate dynamics, and the intrinsic stability of β-barrel proteins.^68,94^

As outlined in Section 2.2, BamA undergoes dynamic transitions between inward-open and lateral-open conformations that drive OMP insertion.^95,96^ These conformational changes are tightly regulated by interactions with accessory lipoproteins (BamCDE) and the POTRA domains, forming a highly adaptable molecular system. Rather than acting as a rigid scaffold, BamA functions as a dynamic machine whose structural plasticity is essential for substrate insertion.^30,95^

As described in Section 2.2, the lateral gate (β1–β16 interface) represents the central mechanistic element of BamA-mediated OMP insertion. This region undergoes reversible opening and closing, enabling strand exchange between BamA and substrate proteins.^57,69^ Experimental restriction of lateral gate mobility abolishes BAM function, underscoring its essential role.^55^

Mechanistically, lateral gate opening enables the formation of hybrid-barrel intermediates, where substrate β-strands interact with BamA *via* β-augmentation.^97,98^ This interaction stabilizes folding intermediates through hydrogen bonding and lowers the energetic cost of inserting polar peptide backbones into the hydrophobic membrane.^64^ In parallel, BAM induces localized membrane thinning and distortion near the lateral gate, further reducing insertion barriers.^59,97^

Once nucleation occurs, β-barrel folding proceeds spontaneously, driven by the strong thermodynamic stability of inter-strand hydrogen bonding within the barrel structure.^99^ Thus, BAM acts as a catalytic scaffold that lowers kinetic barriers and guides folding along a favorable energy landscape rather than directly supplying energy.

From a drug design standpoint, this mechanism highlights several critical principles. First, BamA should be targeted as a dynamic system rather than a static structure, with inhibitors designed to stabilize inactive or non-productive conformations. Second, disruption of lateral gate opening or β-strand augmentation can effectively block OMP insertion. Third, small molecules or peptidomimetics that mimic substrate backbone interactions may competitively inhibit hybrid-barrel formation. Finally, compounds that interfere with membrane remodeling near the lateral gate may further increase the energetic barrier to insertion.

Although some studies suggest that smaller OMPs may exhibit partial independence from lateral gating under specific conditions^100^ the overall process remains fundamentally dependent on BamA dynamics and thermodynamic coupling.

### 3.3 Overall mechanistic–medicinal chemistry integration

Collectively, OMP biogenesis proceeds through a tightly integrated sequence of events: Sec-mediated translocation, chaperone-assisted stabilization, substrate recognition by BAM, POTRA-mediated engagement, and energy-independent membrane insertion driven by BamA dynamics.

This coordinated pathway not only ensures efficient β-barrel assembly but also exposes multiple pharmacologically targetable steps. In particular, the dependence on conformational flexibility, transient protein–protein interactions, and β-strand templating provides a strong foundation for rational inhibitor design. Targeting these dynamic and mechanistically essential features offers a promising strategy for the development of novel antibiotics against Gram-negative pathogens.

### 3.4 Conformational dynamics and SAR principles

The lateral gate of BamA constitutes the primary pharmacological hotspot within the BAM. This region functions as a transient binding interface that mediates substrate engagement through β-strand pairing interactions, a mechanism that is central to OMP insertion.^101,102^ At this interface, substrate recognition is driven by a combination of backbone hydrogen bonding, hydrophobic side-chain contacts, and precise conformational complementarity between the interacting β-strands.^71,103^ From a medicinal chemistry perspective, these features impose stringent design requirements on small-molecule or peptidomimetic inhibitors. Effective BamA inhibitors must accurately mimic the native β-strand geometry and reproduce the characteristic hydrogen-bonding patterns of the lateral gate, while maintaining sufficient conformational rigidity to minimize entropic penalties upon binding.^71^

## 4 BamA as a drug target and inhibitor landscape

### 4.1 Target validation and biological significance

BamA has emerged as a highly validated antibacterial target owing to its essential role in OM biogenesis and β-barrel protein assembly in Gram-negative bacteria. Extensive genetic and biochemical studies have demonstrated that disruption of BamA function leads to severe defects in OM integrity and ultimately bacterial cell death. In *Escherichia coli*, BamA is indispensable, with gene deletion resulting in lethality,^68,90^ while similar essentiality has been confirmed in *Neisseria* species, underscoring its conserved and non-redundant function across Gram-negative pathogens.^104–106^ Cross-species complementation studies further indicate that BamA function is highly specific, reflecting strict structural and evolutionary constraints.^107^

Mechanistic insight has been reinforced through mutational analyses. Conditional mutants with reduced BamA expression (*e.g.*, bamA101) exhibit impaired growth, compromised membrane integrity, and increased sensitivity to BamA-targeting agents.^34^ Conversely, gain-of-function mutations (*e.g.*, E470K, E780D) that alter conformational dynamics can partially compensate for defects in other BAM components, highlighting BamA as the central functional hub of the complex.^108,109^ At the cellular level, inhibition or depletion of BamA results in global defects in OMP folding and insertion, leading to protein misfolding, increased membrane permeability, and collapse of barrier function.^110^ Furthermore, BamA is required for the assembly of key OM proteins such as LptD, amplifying the downstream consequences of its inhibition on bacterial viability.^111^

Pharmacological and immunological studies further support its druggability. Monoclonal antibodies targeting extracellular loops of BamA inhibit OMP folding and induce bacterial death,^112^ while peptides, macrocycles, and small molecules similarly disrupt BamA function and produce lethal envelope defects.^55,113^ Collectively, these findings establish BamA as a structurally unique, surface-accessible, and essential target with a direct link between inhibition and bactericidal activity.

### 4.2 Darobactins and β-strand-mimetic macrocycles

The discovery of darobactins represents a major milestone in BamA-targeted antibiotic development, providing the first highly selective inhibitors of the BAM complex.^114^ Darobactins are ribosomally synthesized bicyclic heptapeptides that bind directly to the lateral gate (β1–β16 interface), the key substrate interaction site described in Section 2.2.^114,115^ This mechanism effectively blocks β-signal recognition, a critical step in OMP folding.^116^

A defining feature of darobactins is their ability to mimic a β-strand, exploiting the β-augmentation mechanism described in Section 3.1.^114,117^ This molecular mimicry allows darobactins to competitively occupy the substrate-binding site and inhibit OMP insertion.

SAR studies have identified conformational rigidity and hydrophobic patterning as primary determinants of activity. The rigid macrocyclic architecture, enforced by intramolecular crosslinks, is essential for maintaining the bioactive conformation required for high-affinity binding.^114,118^ Hydrophobic residues play a key role in engaging membrane-proximal regions, while C-terminal modifications significantly enhance potency by optimizing interactions with hydrophobic pockets near the lateral gate.^119,120^ Incorporation of noncanonical amino acids, including halogenated residues, further improves binding affinity, metabolic stability, and antibacterial activity.^120,121^ Advanced derivatives such as D22 and D69 exemplify how fine-tuning of these features can enhance potency against multidrug-resistant Gram-negative bacteria.^119,122^ Collectively, darobactins establish β-strand mimicry as a powerful and generalizable design principle for BamA inhibition.

These findings suggest that conformational preorganization, rather than mere binding affinity, is the dominant driver of activity, positioning darobactins as prototypical examples of structure-enabled inhibition where β-strand mimicry and rigidity act synergistically to achieve high selectivity.

### 4.3 Expanding landscape of BamA inhibitors

Beyond darobactins, the landscape of BamA-associated antibacterial agents includes peptide-based, macrocyclic, and small-molecule compounds with markedly different levels of target validation. Compounds supported by structural, biochemical, or genetic evidence for direct BamA engagement should be distinguished from phenotypic hits whose effects on outer-membrane biogenesis or integrity have been observed without definitive evidence of direct BamA binding. This distinction is particularly important for small molecules, for which outer-membrane disruption may produce phenotypes that overlap with those expected from BamA inhibition.^123,124^

Among peptide and macrocyclic inhibitors, compounds such as PTB1 and PTB2 exhibit conformation-specific binding behavior, selectively stabilizing distinct functional states of BamA and illustrating the principle of state-dependent inhibition.^29^ Similarly, JB95 and related macrocyclic peptides, including peptide 3, modulate lateral gate dynamics, while lumen-binding peptides (CP1–CP3) interfere with later stages of OMP assembly by targeting the internal cavity of the β-barrel.^34^ Collectively, these agents demonstrate that effective BamA inhibition can be achieved through modulation of its conformational landscape rather than simple occupancy of a single binding site.

Advances in synthetic and display technologies have further enabled the discovery of macrocyclic peptides capable of engaging gate-adjacent regions and lumen-facing subpockets, stabilizing inactive conformations and revealing previously unrecognized binding sites within BamA.^34^ These findings support a model in which transient and cryptic structural features can be exploited for inhibitor design.

Among the reported small-molecule agents, MRL-494 and IMB-H4 have been investigated as BamA-associated modulators, whereas VUF15259 and nitazoxanide have primarily been characterized through phenotypic effects on outer-membrane biogenesis and integrity. For VUF15259 and nitazoxanide, definitive structural or biochemical evidence demonstrating direct binding to BamA is currently lacking. Accordingly, these compounds are more appropriately described as phenotypic or BamA-associated hits rather than confirmed direct BamA inhibitors. This distinction is important because outer-membrane damage can arise through mechanisms independent of direct BamA engagement^125^ (Fig. 3).

**Fig. 3:**
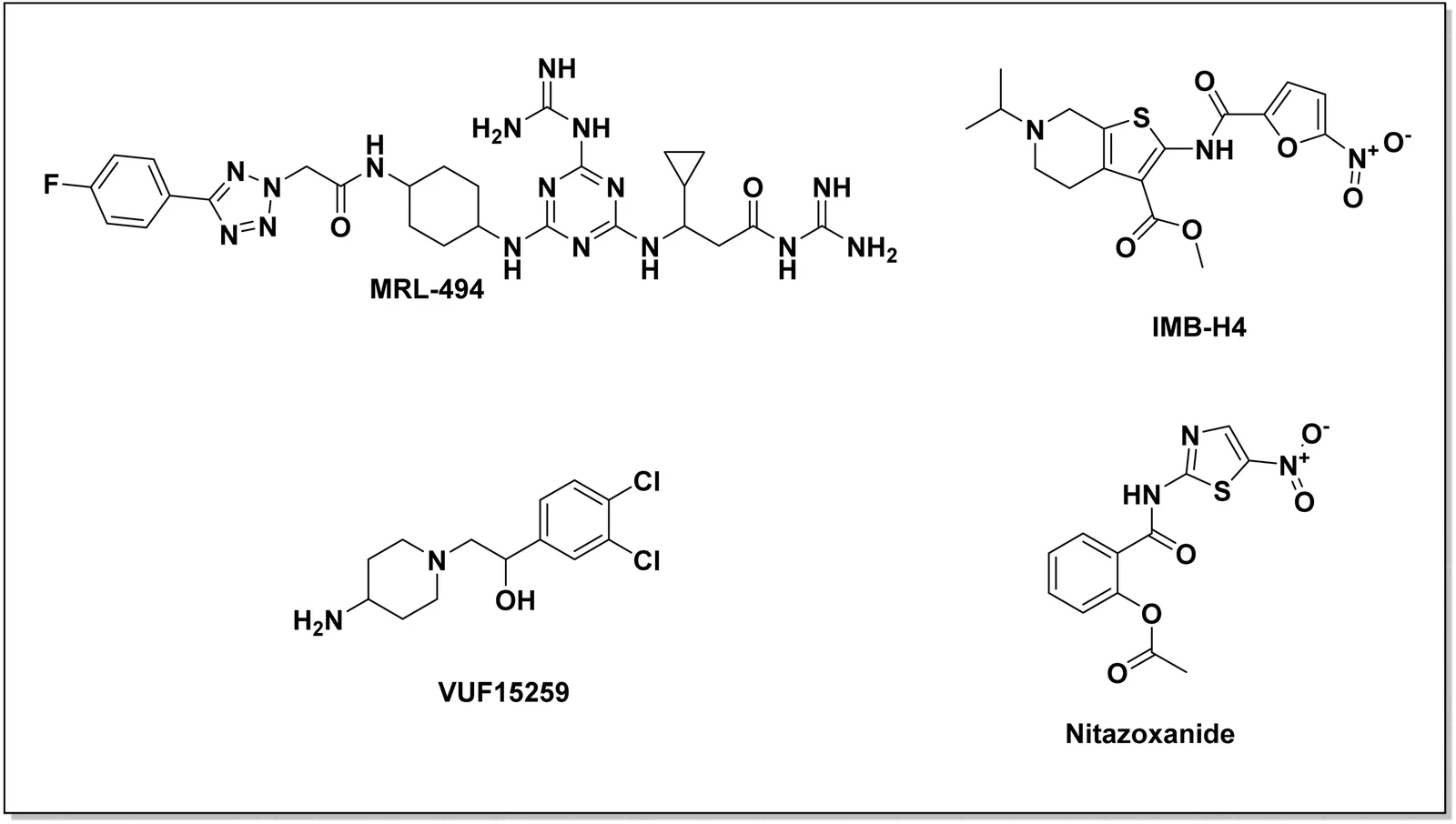
Small-molecule compounds reported to modulate BamA/BAM-associated functions, categorized according to the level of evidence for direct BamA engagement. Compounds are distinguished according to whether BamA involvement is supported by direct structural or biochemical evidence or inferred primarily from phenotypic effects on outer-membrane biogenesis. This distinction is important because disruption of outer-membrane homeostasis does not necessarily establish direct binding to BamA.

Peptidomimetic scaffolds also contribute to this expanding landscape. Polyphor peptide 7, for example, has been shown to impair OM integrity while interfering with BamA-mediated OMP biogenesis and is frequently used as a comparative benchmark in antimicrobial studies.^126^ This dual activity illustrates the functional interplay between membrane destabilization and BAM inhibition.

Overall, these findings establish BamA as a conformationally adaptable and multi-site target, where inhibitor efficacy is governed by the ability to modulate dynamic structural states. The mechanistic features of emerging natural product scaffolds and the design implications of these findings are discussed in the following sections.

### 4.4 Emerging natural product scaffolds and peptidomimetics

While Section 4.3 provides a broad overview of the diverse classes of BamA inhibitors, the following section focuses specifically on emerging natural product scaffolds and peptidomimetics, highlighting their unique mechanistic features and implications for inhibitor design.

Building on the broader inhibitor landscape described above, recent efforts have focused on the identification of structurally diverse natural product scaffolds and engineered peptidomimetics that expand the chemical space of BamA-targeting agents. Among these, xenorceptides and bamabactins represent distinct peptide families that function as non-canonical β-strand mimetics with unique binding geometries.^27^

Mechanistically, these compounds engage the lateral gate region through β-augmentation-like interactions but do not strictly replicate native substrate β-strand architecture. Instead, they induce conformational perturbations that disrupt the coordinated process of OMP folding and insertion. This mode of action highlights the importance of conformational control in BamA function and demonstrates that effective inhibition can be achieved through structural distortion rather than direct competition with substrate binding.

From a medicinal chemistry perspective, these scaffolds provide important insights into the design of next-generation inhibitors. In particular, they demonstrate that strict backbone replication is not a prerequisite for activity, and that alternative backbone conformations and side-chain arrangements can achieve productive binding. This expands the design space beyond classical substrate mimicry and enables the exploration of structurally diverse peptidomimetic frameworks.

Overall, emerging natural product scaffolds reinforce the concept that structural diversity and conformational adaptability are key determinants of BamA inhibition, facilitating the transition from naturally derived compounds to rationally optimized therapeutic candidates.

### 4.5 Small-molecule inhibitors: design challenges and opportunities

In contrast to the peptide-based and natural product scaffolds discussed above, this section examines the distinct challenges associated with small-molecule targeting of BamA, with an emphasis on design limitations and future opportunities.

In contrast to peptide-based scaffolds, the development of small-molecule inhibitors targeting BamA presents significant challenges. The protein lacks a well-defined catalytic pocket and instead features large, dynamic, and relatively shallow interaction surfaces, which limit the ability of conventional small molecules to achieve high-affinity and selective binding.

Compounds such as MRL-494 and IMB-H4 illustrate both the potential and limitations of this approach. These molecules demonstrate that BamA function can be perturbed through interactions with extracellular regions or by disrupting protein–protein interactions within the BAM complex. However, their antibacterial activity is often associated with challenges related to specificity, OM permeability, and off-target membrane effects.^125^

These limitations emphasize the need for alternative design strategies. Approaches such as targeting transient or allosteric binding pockets, incorporating peptidomimetic features, and exploiting protein–protein interaction interfaces may improve binding efficiency and selectivity. In particular, hybrid molecules that integrate structural elements of peptides with drug-like small-molecule properties represent a promising direction for future development.

Overall, successful small-molecule targeting of BamA will likely require moving beyond traditional paradigms toward strategies that account for the dynamic and structurally complex nature of the protein. Table 1 provides a mechanistic comparison of BamA-targeting and BamA-associated compounds according to their binding site, mode of action, and level of evidence for direct BamA engagement. Because quantitative antibacterial activity and *in vivo* efficacy data have not been reported uniformly across these chemically and mechanistically diverse compounds, the table focuses on parameters that permit consistent comparison without implying direct equivalence of potency across different experimental systems.

**Table 1:** Mechanistic and activity comparison of BamA-targeting and BamA-associated compounds according to binding site, mechanism of action, evidence for BamA engagement, reported minimum inhibitory concentration (MIC/MIC_90_), and available *in vivo* efficacy

Compound	Binding site	Mechanism of action	Evidence for BamA engagement	Reported MIC/MIC_90_ (µg mL; µM where reported)	*In vivo* efficacy
Darobactin A	Lateral gate (β1–β16)	β-Strand mimicry	Direct structural and genetic evidence	2 µg mL^−1^ (*E. coli* ATCC 25922)^127^	Yes; mouse septicemia models^127^
Darobactin D22	Lateral gate (β1–β16)	β-Strand mimicry and conformational trapping	Structural and biochemical evidence	MIC_90_: 2–4 µg mL^−1^ (*E. coli*); 4–8 µg mL^−1^ (*K. pneumoniae*); 8–16 µg mL^−1^ (*P. aeruginosa*); 8 µg mL^−1^ (*A. baumannii*)^128^	Yes; murine *P. aeruginosa* thigh-infection, *E. coli* peritonitis/sepsis and urinary-tract infection models; zebrafish *A. baumannii* infection model^128^
Dynobactin A	Lateral gate/lumen	Conformational trapping	Direct structural evidence	8 µg mL^−1^ (*E. coli* ATCC 25922); 16 µg mL^−1^ (*E. coli* K-12 MG1655)^40^	Yes; mouse systemic *E. coli* infection model^40^
PTB1	Conformation-specific site	State-dependent inhibition	Structural and biochemical evidence	42 µg mL^−1^ (25 µM) (*E. coli*)^29^	
PTB2	Conformation-specific site	State-dependent inhibition	Structural and biochemical evidence	4 µg mL^−1^ (2 µM) (*E. coli*)^29^	
JB-95	Lateral gate/outer-membrane-associated region	Gate modulation and outer-membrane disruption	Target-associated evidence	∼0.25 µg mL^−1^ (*E. coli*)^129^	
Peptide 3	BamA extracellular loops	BAM/outer-membrane interference	Structural and functional evidence	0.5 µg mL^−1^ (*E. coli*)^130^	
CP1–CP3	BamA β-barrel lumen	Inhibition of OMP assembly	Direct binding and functional evidence	∼6.3–12.5 µM in BAM-compromised *E. coli* strains; no inhibitory concentration reported for WT *E. coli*^131^	
Bamabactins	Lateral gate	β-Strand mimicry	Structural and target-binding evidence		
Xenorceptides	Lateral gate	Conformational disruption	Mechanistic and structural evidence		
MRL-494	Extracellular region	BamA-associated functional disruption	Biochemical and genetic evidence	8–32 µg mL^−1^ in susceptible tested strains; >128 µg mL^−1^ (*K. pneumoniae*)^132^	
IMB-H4	BamA–BamD interface	Protein–protein interaction disruption	Biochemical and target-associated evidence	4 µg mL^−1^ (*E. coli* ATCC 25922); 4–32 µg mL^−1^ (clinical *E. coli* isolates)^133^	
VUF15259	Uncertain	OM/BAM perturbation	Phenotypic evidence; direct BamA binding unconfirmed		
Nitazoxanide	Uncertain	OM biogenesis disruption	Phenotypic evidence; direct BamA binding unconfirmed		
Licochalcone A	BamA/BamD-associated	OM/BAM modulation	Biochemical interaction evidence		
Polyphor peptide 7	OM/BamA-associated	Membrane disruption and BAM interference	Mixed/limited target specificity	0.25 µg mL^−1^ (*E. coli*)^134^	

Blank cells indicate that a corresponding quantitative MIC/MIC_90_ or *in vivo* efficacy value was not identified in the publicly available experimental literature reviewed for this table. Reported MIC values are organism- and strain-specific and should not be interpreted as directly comparable measures of potency across all compounds. For CP1–CP3, the reported inhibitory concentrations apply to BAM-compromised *E. coli* strains rather than wild-type *E. coli*.

### 4.6 Biologics and alternative therapeutic modalities

Biologics and alternative modalities provide complementary strategies for BamA inhibition. Monoclonal antibodies targeting extracellular loops have been shown to effectively block OMP folding and induce bacterial death, confirming the accessibility of BamA on the bacterial surface.^112^

In addition, bacteriocins such as LlpA and bacteroidetocins exploit naturally evolved mechanisms to selectively target BamA, while engineered peptides and peptidomimetics can disrupt critical protein–protein interactions within the BAM complex.^126^ These approaches highlight the versatility of BamA as a therapeutic target and underscore the potential of non-traditional modalities in combating Gram-negative infections.

These approaches highlight that BamA is uniquely amenable to large-molecule targeting, further supporting its accessibility and reinforcing the potential of non-traditional modalities in overcoming Gram-negative permeability barriers.

Fig. 4 summarizes the integrated mechanistic and SAR landscape of BamA inhibitors. Key SAR principles emphasize the importance of conformational rigidity, β-strand mimicry, backbone hydrogen bonding, and hydrophobic patterning for high-affinity binding, while small-molecule approaches remain constrained by permeability and shallow binding interfaces. Collectively, the aforementioned inhibitors converge on blocking β-barrel assembly, leading to OM failure and bacterial cell death.

**Fig. 4:**
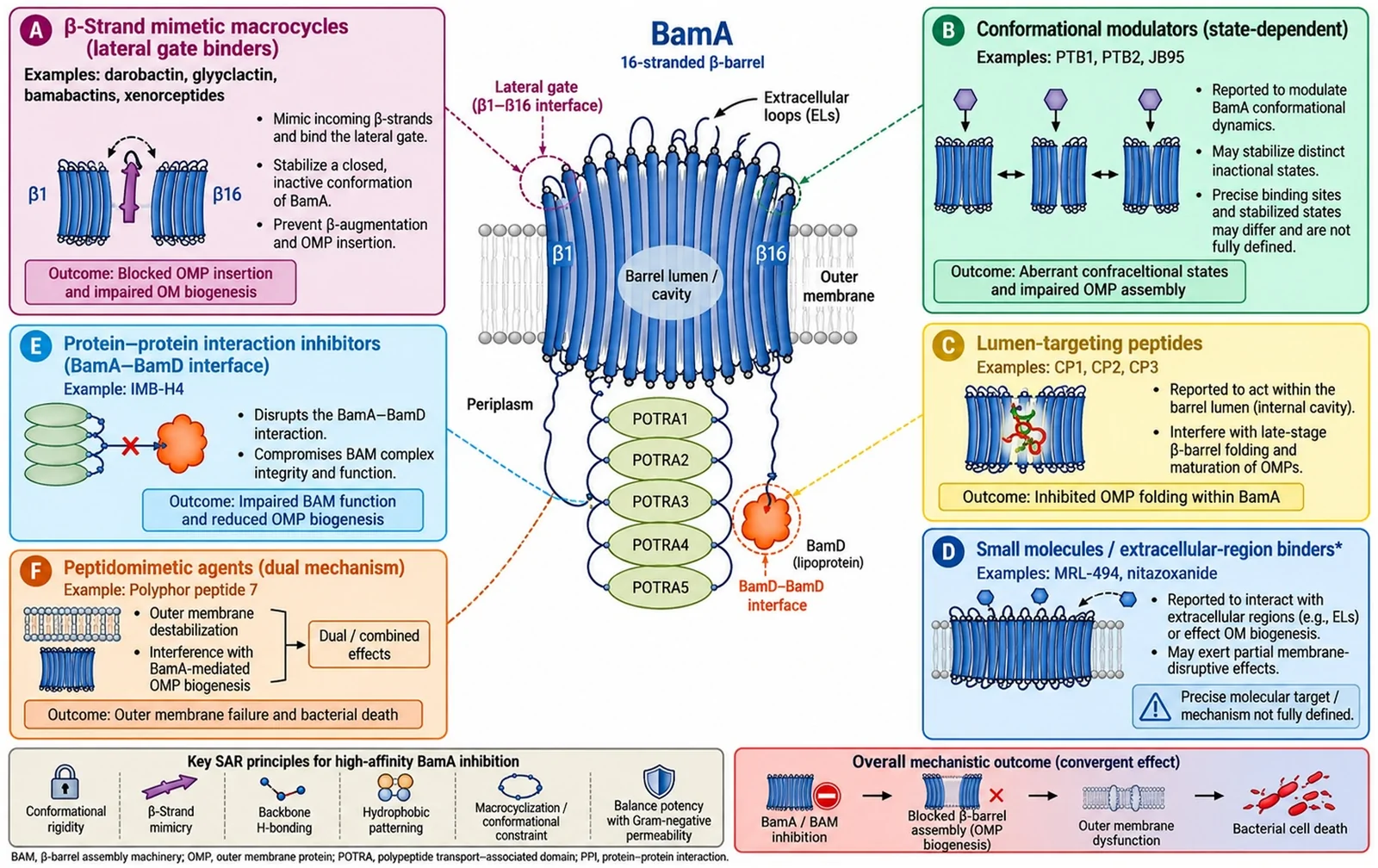
Integrated mechanistic and SAR landscape of BamA inhibitors. Major inhibitor classes exploit distinct structural and functional features of BamA, including (A) the lateral gate through β-strand-mimetic macrocycles, (B) conformationally sensitive regions through state-dependent conformational modulators, (C) the β-barrel lumen through lumen-targeting peptides, (D) extracellular regions through small-molecule/extracellular-region binders, (E) the BamA–BamD protein–protein interaction interface through protein–protein interaction inhibitors, and (F) dual mechanisms through peptidomimetic agents. Key medicinal chemistry principles include β-strand mimicry, backbone-mediated hydrogen bonding, conformational rigidity, hydrophobic patterning, macrocyclization, and modulation of BamA conformational dynamics. Despite differences in scaffold, binding site, and mechanism, these strategies converge on disruption of BamA-mediated OMP folding and outer-membrane biogenesis, ultimately compromising bacterial viability (generated with NanoBanana).

### 4.7 Critical medicinal chemistry perspective and future directions

The BamA inhibitor landscape reflects a clear shift from single-mechanism inhibition toward mechanistic and structural diversification. While β-strand mimicry, as mechanistically outlined in Section 3.1, remains a central design principle for BamA inhibition, demonstrate that effective inhibition can be achieved through conformational trapping, multi-site engagement, and disruption of protein–protein interactions.

A key insight is that modulation of BamA conformational dynamics (see Section 2.2) represents a highly effective strategy for inhibition. This aligns with broader trends in drug discovery, where targeting protein dynamics is increasingly recognized as a critical determinant of activity.

Balancing structural complexity with drug-like properties remains a central challenge. While macrocyclic peptides offer high potency and selectivity, their pharmacokinetic limitations must be addressed, whereas small molecules require improved strategies to achieve sufficient affinity and specificity. Hybrid peptidomimetic approaches may provide a viable solution to bridge this gap.

Finally, the ability to target multiple functional regions of BamA offers a promising avenue for overcoming antibiotic resistance. By exploiting diverse mechanisms and binding sites, next-generation inhibitors may achieve improved durability against resistance mutations. Continued integration of structural biology, computational modeling, and SAR-driven optimization will be essential to fully realize the therapeutic potential of BamA-targeted agents.

Taken together, these observations suggest that future BamA inhibitors will likely emerge from strategies that integrate conformational control, multi-site targeting, and hybrid scaffold design, rather than relying on single-mechanism approaches.

### 4.8 Resistance liability and evolutionary constraints of BamA targeting

An important consideration in evaluating BamA as an antibacterial target is its intrinsic susceptibility to resistance development. Unlike many classical antibiotic targets, BamA operates under strong evolutionary and structural constraints that significantly restrict the range of mutations compatible with sustained bacterial viability. These constraints arise from BamA's essential and non-redundant role in OM biogenesis, its high degree of conservation across Gram-negative species, and the extensive overlap between inhibitor-binding surfaces and native substrate recognition interfaces.

BamA belongs to the Omp85 protein family and is highly conserved across Gram-negative bacteria, reflecting its indispensable function in β-barrel OMP assembly. In particular, the lateral gate region (β1–β16 interface) remains structurally conserved due to its central role in substrate recognition and membrane insertion. This conservation restricts the tolerance for mutations within this region without compromising protein function.^135,136^

A defining feature of BamA-targeting inhibitors, exemplified by darobactins, is their exploitation of native substrate recognition mechanisms. These compounds act as β-strand mimetics, engaging the substrate recognition mechanism discussed in Section 3.1.^108^ Consequently, the inhibitor-binding interface coincides with a functionally essential surface required for OMP biogenesis.^101,114^

This functional overlap imposes a significant evolutionary constraint. Mutations that reduce inhibitor binding often simultaneously impair substrate recognition, lateral gate dynamics, or β-barrel templating. Computational and experimental studies indicate that resistance-associated substitutions typically alter conformational flexibility rather than abolish binding entirely, thereby preserving partial function while reducing inhibitor affinity.^95,101^

In contrast to classical intracellular targets that tolerate compensatory mutations, BamA lacks redundancy and does not possess a discrete catalytic pocket. Even modest perturbations in its conformational dynamics can propagate through the BAM complex, leading to global defects in OM protein folding and membrane integrity.^135,136^

Experimental observations support the existence of functional constraints associated with resistance-associated BamA mutations. Such mutations, particularly those affecting the lateral gate and adjacent extracellular loops, have been associated with reduced growth, impaired virulence, and diminished competitive fitness 130. However, these fitness effects should be interpreted cautiously because much of the available evidence is derived from controlled *in vitro* growth, competition, or experimental infection systems that may not fully reproduce the complex selective pressures encountered during natural infection. Host-associated factors, including nutrient availability, immune pressure, tissue-specific conditions, and interactions with microbial communities, may alter the relative fitness of resistant populations. Moreover, compensatory mutations or other adaptive changes can partially restore fitness while retaining the resistance phenotype, as documented broadly for antibiotic resistance evolution. Therefore, the fitness costs observed for BamA resistance mutations should be regarded as important evolutionary constraints, but not as absolute barriers to the persistence or further evolution of resistance.^127,137,138^

Collectively, BamA represents a structurally constrained antibacterial target in which resistance can emerge and may initially carry substantial functional costs; however, the extent to which these costs limit resistance persistence *in vivo* remains uncertain because compensatory adaptation and host-associated selection may partially offset fitness defects.

### 4.9 Clinical translation and development challenges

Despite significant advances in the identification and optimization of BamA inhibitors, their translation into clinically viable antibiotics remains at an early stage. Darobactins represent the most extensively characterized example, demonstrating potent activity against multidrug-resistant Gram-negative pathogens and efficacy in multiple animal infection models. However, progression from proof-of-concept efficacy to clinical development requires simultaneous optimization of pharmacokinetic, manufacturing, safety, and administration properties.^128,139^

A major translational limitation of macrocyclic peptide inhibitors is their pharmacokinetic profile. Although conformational constraint and post-translational modification can improve stability relative to linear peptides, systemic exposure remains an important consideration for darobactin development. Recent pharmacokinetic studies of the optimized darobactin derivative D22 demonstrated a biphasic elimination profile following intravenous administration, with a predominant plasma half-life of approximately 0.6 h. Oral bioavailability was negligible, with an estimated bioavailability of only 0.62%, whereas subcutaneous and intratracheal administration produced measurable systemic or pulmonary exposure. These properties constrain conventional oral dosing and highlight the need to improve systemic persistence and exposure while preserving BamA affinity.^128^

Importantly, the short *in vivo* half-life of D22 does not appear to result simply from rapid metabolic degradation. *In vitro* ADMET studies showed that D22 and D69 were highly stable in mouse, rat, and human liver microsomes and plasma, with no substantial degradation over the tested periods. Instead, their physicochemical properties, including high polarity and relatively low plasma protein binding, together with rapid systemic disposition, may contribute to the unfavorable *in vivo* pharmacokinetic profile. These findings indicate that further structural optimization should focus not only on metabolic stability but also on physicochemical properties governing systemic exposure and clearance.^140^

The large and structurally complex nature of darobactins also creates challenges for manufacturing and scalable production. Their unusual bicyclic architecture contains two distinctive macrocyclic crosslinks and requires noncanonical amino-acid building blocks, creating substantial synthetic and stereochemical challenges. Although heterologous biosynthetic production has enabled access to darobactin analogues, total chemical synthesis has also been developed to facilitate material supply and structural diversification. Nevertheless, scalable, reproducible, and cost-effective production remains an important consideration for future development, particularly when extensive structural optimization and formulation studies require reliable access to complex analogues.^141,142^

Administration represents another important bottleneck. The negligible oral bioavailability observed for D22 limits its suitability for conventional oral therapy and currently favors parenteral or locally targeted approaches for systemic or site-specific infections. Notably, intratracheal administration produced measurable epithelial lining fluid and lung exposure, while subcutaneous administration provided systemic exposure and substantial kidney distribution. These findings indicate that the administration route can substantially influence the pharmacological profile of darobactins and should therefore be considered during lead optimization rather than treated solely as a formulation issue.^128^

Safety and immunological considerations also require further investigation as development progresses. Available preclinical studies of D22 and D69 have reported favorable cytotoxicity and safety profiles, including no detectable cytotoxicity in HepG2 cells and favorable tolerability in the reported animal studies. However, these findings do not eliminate the need for comprehensive evaluation of repeated-dose toxicity, immunogenicity, off-target interactions, and host-tissue effects during subsequent development. Thus, immunological and safety liabilities should be considered important developmental questions, rather than established barriers specific to darobactins.^128,140^

For darobactins specifically, the major translational challenge is therefore not target validation or antibacterial potency, both of which are strongly supported, but achieving a clinically appropriate balance between potency, systemic exposure, stability, manufacturing feasibility, and practical administration. Structure-guided optimization and biosynthetic engineering have already generated derivatives with improved antibacterial activity, while total synthesis provides complementary opportunities for structural diversification. Nevertheless, further pharmacokinetic optimization, formulation development, safety assessment, and translational pharmacology will be required before these compounds can progress from promising preclinical candidates toward clinical evaluation.^140,143^

## 5 Resistance mechanisms and synergistic strategies

Although BamA is an evolutionarily conserved and structurally constrained component of the BAM complex, resistance to BamA-targeting agents such as darobactins can still emerge under selective pressure. However, both the nature of resistance mutations and the functional importance of BamA significantly limit their evolutionary persistence.

### 5.1 Resistance mechanisms and evolutionary constraints

Resistance to BamA-targeting agents, particularly darobactins, primarily arises through localized point mutations within the BamA protein. These mutations are predominantly clustered in extracellular loops surrounding the lateral gate (β1–β16 interface), which constitutes the principal binding region for β-strand mimetic inhibitors. Reported substitutions include G429V, T434A, E435K, G443D, Q445P, F394V, G807V, and A705T, among others.^68^

Mechanistically, these mutations modulate the conformational dynamics of the lateral gate, often by increasing its flexibility or altering its opening frequency. Such changes reduce inhibitor binding affinity by destabilizing key interactions required for effective β-strand mimicry, thereby decreasing inhibitor residence time while preserving partial protein function.^71,97^

Despite this, darobactins retain a degree of resilience to resistance due to their β-augmentation binding mode, which relies predominantly on backbone-mediated hydrogen bonding rather than strict side-chain complementarity.^114^ As a result, single-point mutations typically produce only modest reductions in susceptibility compared to conventional small-molecule inhibitors.^144^

Importantly, resistance-associated mutations impose significant functional constraints. Alterations in the lateral gate region can compromise OMP folding efficiency, leading to defects in membrane integrity and reduced bacterial fitness. Consistent with this, resistant strains often exhibit attenuated growth, impaired virulence, and decreased competitive fitness.^145^

Nevertheless, the evolutionary consequences of these fitness costs may differ between controlled laboratory conditions and host-associated environments. *In vivo*, resistant bacteria are exposed to heterogeneous nutrient availability, immune-mediated pressures, tissue-specific physicochemical conditions, and competition with other microorganisms, all of which can modify the relative fitness of resistance mutants. In addition, compensatory mutations or epistatic adaptations at secondary loci may partially restore growth, survival, or virulence without eliminating the resistance phenotype. Consequently, the fitness penalties associated with BamA resistance mutations may be reduced during prolonged host-associated selection, potentially allowing resistant populations to persist or evolve further despite an initial fitness disadvantage. This possibility remains insufficiently characterized for BamA and represents an important limitation of current resistance-evolution studies.^137,138,146^

From a medicinal chemistry perspective, these findings suggest that BamA inhibitors should favor backbone-mediated interactions over strict side-chain recognition, particularly within conserved regions of the lateral gate. Because the gate undergoes substantial conformational movement, some degree of structural adaptability may also be advantageous. In addition, engaging more than one functional region of BamA could reduce the likelihood of mutational escape and improve the robustness of inhibition.

Collectively, resistance to BamA inhibitors is mechanistically feasible and may be constrained by the essential and structurally sensitive nature of BamA; however, these constraints should not be considered absolute, as compensatory adaptation and host-associated selection could partially mitigate the fitness costs of resistance and influence its persistence *in vivo*.

### 5.2 Synergistic and combination strategies

Given the OM permeability barrier in Gram-negative bacteria, combination strategies that enhance drug penetration and amplify the effects of BamA inhibition represent a promising approach to improve antibacterial efficacy and limit resistance emergence.

One of the most effective strategies involves combining BamA inhibitors with OM-disrupting agents, particularly those targeting LPS architecture such as polymyxins. These agents bind lipid A and disrupt cation-mediated stabilization of the OM, increasing membrane permeability and facilitating access of BamA-targeting compounds to their sites of action.^147,148^ Enhanced permeability promotes intracellular accumulation and can reduce the impact of efflux mechanisms.^149,150^

BamA inhibition further reinforces this effect by impairing OMP assembly, contributing to global destabilization of the OM and amplifying antibacterial activity.^68,112,151^ This reciprocal interaction creates a synergistic cycle in which membrane disruption enhances inhibitor access, while BamA inhibition exacerbates membrane dysfunction.

Beyond polymyxins, synergy has been observed with multiple antibiotic classes, including β-lactams,^152–154^ fosfomycin,^129^ and hydrophobic antibiotics with poor intrinsic permeability.^155^ Non-antibiotic adjuvants such as phenothiazines, cannabidiol, and fusidic acid have also shown synergy *via* permeability enhancement mechanisms.^156–158^

Despite these advantages, combination strategies require careful optimization. Suboptimal dosing or imbalanced regimens may promote the selection of partially resistant populations.^159^ Therefore, pharmacodynamic optimization is essential to maximize bactericidal synergy while minimizing the risk of resistance development.

#### 5.2.1 Integrated perspective

Together, current evidence supports a unified model in which resistance to BamA inhibitors arises primarily through mutations in the lateral gate region but remains limited by associated fitness costs and structural constraints.

At the same time, synergistic strategies enhance antibacterial efficacy by increasing OM permeability and facilitating access to BamA, while inhibition of OMP assembly further destabilizes membrane integrity. This reciprocal reinforcement between membrane disruption and BamA inhibition amplifies antibacterial activity.

Thus, although resistance can occur at the molecular level, its evolutionary sustainability is restricted. In contrast, rationally designed combination therapies provide a powerful approach to enhance efficacy, overcome permeability barriers, and suppress resistance development in Gram-negative pathogens^123,160,161^ (Fig. 5).

**Fig. 5:**
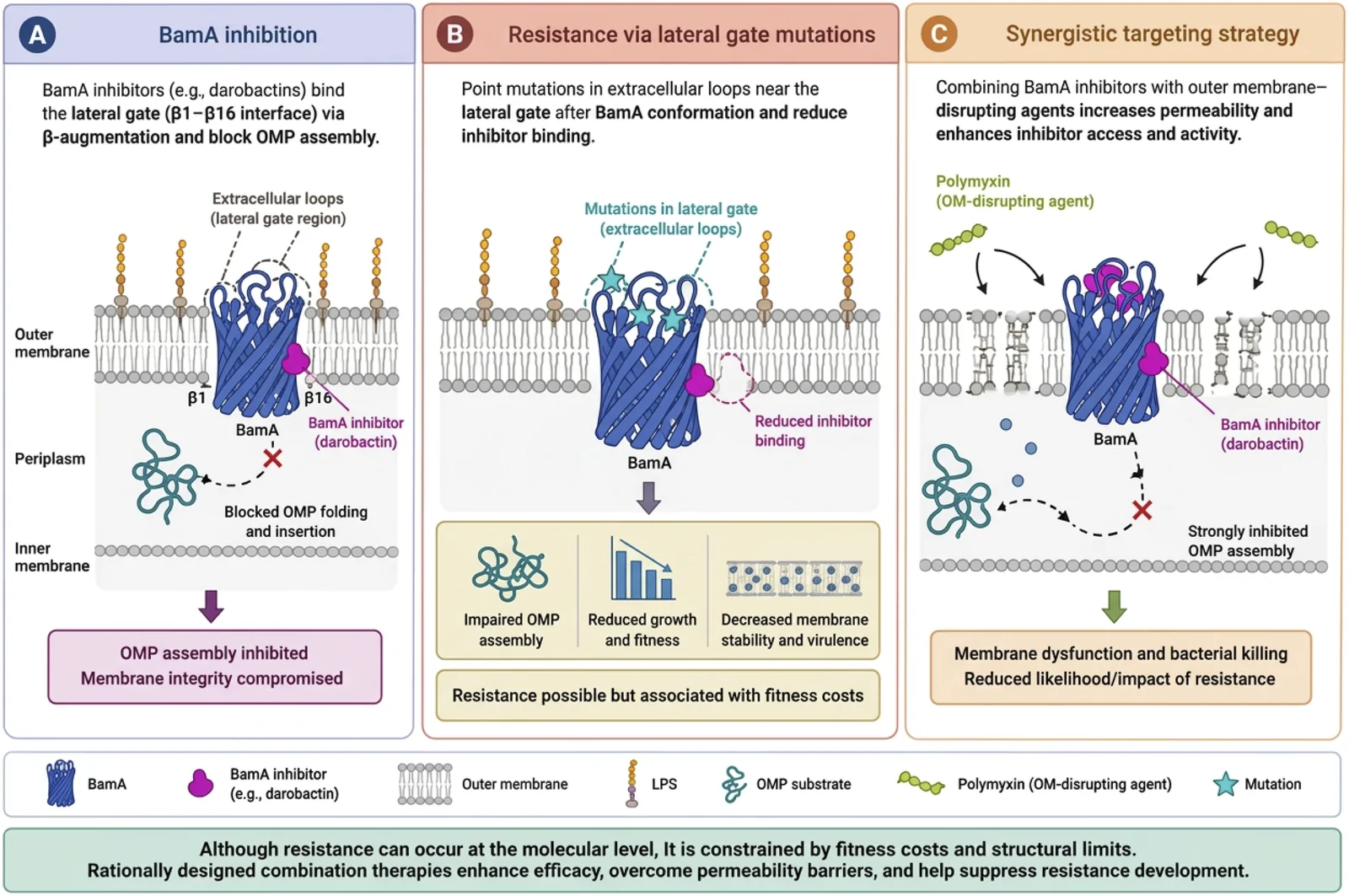
Resistance mechanisms and synergistic targeting strategies of BamA inhibitors in Gram-negative bacteria. (A) BamA inhibition: BamA-targeting agents, exemplified by darobactin, bind the lateral gate at the β1–β16 interface, prevent β-augmentation, and interfere with OMP assembly and folding. (B) Resistance *via* lateral-gate mutations: mutations in the extracellular loops surrounding the lateral gate can alter BamA conformation and reduce inhibitor binding, potentially compromising antibacterial activity while imposing associated fitness and membrane-function costs. (C) Synergistic targeting strategy: combining BamA inhibitors with outer-membrane-disrupting agents, such as polymyxins, can increase membrane permeability and inhibitor access, thereby enhancing BamA inhibition and OMP-assembly defects. These complementary mechanisms may improve antibacterial efficacy and reduce the likelihood of resistance emergence, although the persistence and biological relevance of resistance-associated fitness costs under *in vivo* conditions require further investigation (generated with NanoBanana).

## 6 Medicinal chemistry and pharmacokinetic challenges

The development of inhibitors targeting BamA is inherently challenging due to its localization in the OM of Gram-negative bacteria and its functional interfaces extending into the periplasm. Compounds designed to engage BamA—particularly at periplasmic sites—must overcome a combination of permeability barriers, active efflux mechanisms, and stringent physicochemical constraints, all of which complicate drug design and optimization.^162^

### 6.1 Outer membrane permeability barrier

Drug discovery efforts against BamA are constrained by several fundamental physicochemical and biological limitations. The OM permeability barrier restricts the entry of many compounds into Gram-negative bacteria, particularly bulky, highly hydrophobic, or highly charged molecules. Even compounds that successfully cross the OM may be subject to active expulsion by bacterial efflux pumps, which can substantially reduce their effective concentration at the target site. In addition, BamA-targeting compounds must operate within a narrow physicochemical space in which molecular size, polarity, lipophilicity, and charge must be carefully balanced to achieve adequate permeability while maintaining favorable drug-like properties. Finally, target accessibility varies according to the location of the binding site within BamA, with extracellular regions generally being more accessible than sites that require penetration of the OM and accumulation within the periplasmic space.

The Gram-negative OM, enriched with LPS, acts as a highly selective permeability barrier that restricts entry of bulky, hydrophobic, or highly charged molecules.^163–165^ This significantly limits intracellular or periplasmic drug accumulation and remains a primary obstacle in antibiotic design.

Even when compounds successfully penetrate the OM, they are frequently subjected to active efflux by resistance-nodulation-division (RND) systems, particularly the AcrAB–TolC pump, which recognizes structurally diverse substrates and efficiently reduces intracellular drug concentration.^166^

As a result, BamA inhibitors must occupy a narrow physicochemical space in which properties such as molecular size, polarity, lipophilicity, and charge distribution are finely balanced. Small deviations in these parameters can dramatically affect permeability, efflux susceptibility, and biological activity.^165,167,168^

### 6.2 Physicochemical property optimization

Successful Gram-negative antibacterials require the simultaneous optimization of multiple interdependent physicochemical properties. Compounds that are highly polar often exhibit poor permeability across the OM, limiting their ability to reach intracellular or periplasmic targets. Conversely, highly lipophilic molecules may become sequestered within the membrane or be rapidly recognized and expelled by efflux systems, reducing their effective concentration. In addition, large or highly charged compounds are typically excluded by porin channels and the tightly organized LPS layer, further restricting their entry. Together, these constraints necessitate a careful balance of molecular properties to achieve optimal permeability, retention, and antibacterial activity.

Therefore, BamA-targeting compounds must achieve a delicate balance between sufficient hydrophilicity for solubility and controlled lipophilicity for membrane traversal. This constrained chemical space significantly limits scaffold diversity and complicates lead optimization.^165,167,168^

### 6.3 Outer membrane permeability and target accessibility

Target accessibility is strongly influenced by the structural location of the binding site within BamA. The protein presents both extracellular and periplasmic druggable regions, each associated with distinct accessibility constraints. Extracellular binding sites, such as the lateral gate region, are relatively more accessible and can be targeted without the need for complete traversal of the OM. In contrast, periplasmic interfaces, including BamA–BamD interactions, require compounds to fully penetrate the OM and subsequently accumulate within the periplasmic space. This difference imposes significantly greater challenges for the design and optimization of inhibitors targeting periplasmic sites.

Certain compounds, such as MRL-494, exploit extracellular binding to bypass permeability constraints entirely.^123,169,170^ In contrast, periplasmic inhibitors face the full combined burden of OM penetration and efflux resistance, making their optimization significantly more challenging.

Additionally, BamA itself exhibits conformational dynamics, including lateral gate opening and membrane-associated structural rearrangements, which influence ligand accessibility and binding kinetics.^59^ These dynamic states further complicate rational design efforts.

### 6.4 Efflux-mediated limitations

Efflux pumps, particularly the AcrAB–TolC system, represent a major determinant of intracellular drug exposure. These systems can actively remove chemically diverse compounds, reducing effective intracellular concentrations even after successful OM penetration.^166^

Therefore, effective BamA inhibitors must be carefully designed to evade efflux-mediated clearance mechanisms. This includes avoiding structural motifs that are readily recognized by efflux systems, particularly RND transporters, and minimizing features that increase susceptibility to active extrusion. In addition, compounds must be optimized to maintain sufficient intracellular retention once uptake has occurred, ensuring that effective concentrations are sustained at the target site.

This often requires careful tuning of molecular polarity, flexibility, and conformational properties.

### 6.5 Macrocycles and the darobactin paradigm

Macrocyclic scaffolds, particularly darobactins, represent a breakthrough class of BamA inhibitors. Darobactins bind the lateral gate of BamA by mimicking β-strand interactions involved in natural substrate recognition, thereby inhibiting OMP assembly.^114,169^

This class offers several key advantages that contribute to its effectiveness as BamA inhibitors. The bicyclic architecture confers a high degree of conformational rigidity, which reduces the entropic penalty upon binding and enhances overall binding efficiency. Additionally, these compounds exhibit high specificity driven by backbone-mediated interactions that closely mimic natural substrate recognition. Importantly, their ability to target extracellular regions of BamA allows them to bypass the need for full OM penetration, thereby overcoming one of the major barriers associated with Gram-negative antibacterial drug design.

Structure-guided optimization has yielded engineered derivatives with up to 128-fold improved potency in some cases.^119,122^

However, despite these advantages, macrocyclic compounds still present several important challenges. Their relatively large size can limit diffusion, particularly when targeting periplasmic sites that require traversal of the OM. In many cases, their uptake may depend on specialized transport pathways, which can restrict their spectrum of activity and consistency of intracellular accumulation. Additionally, inherent size-related constraints can further hinder efficient membrane traversal, complicating their optimization for targets that are not readily accessible from the extracellular environment.

Thus, while macrocycles are highly effective for extracellular targets, additional engineering strategies are required for intracellular or periplasmic inhibition.

### 6.6 Integrated design strategies

Collectively, the successful design of BamA inhibitors requires a comprehensive multi-parameter optimization strategy. This involves carefully fine-tuning molecular properties such as polarity, lipophilicity, and size to enable efficient penetration of the OM, while simultaneously minimizing susceptibility to efflux through informed structural design. In addition, leveraging uptake pathways, including porin-mediated entry, can enhance intracellular access. Whenever feasible, prioritizing extracellular BamA binding sites can help circumvent permeability barriers. Incorporating conformational constraints is also critical to improve binding affinity and selectivity, and the use of macrocycles or other rigid scaffolds can further strengthen target engagement.

These principles collectively define a highly restricted but exploitable chemical space for BamA-targeted antibiotic development.

### 6.7 Limitations and current research gaps

Despite significant advances in the field, several critical limitations remain. Much of the current mechanistic understanding is derived from *in vitro* and structural studies, which may not fully capture the complexity of the bacterial OM *in vivo*. Furthermore, most BamA inhibitors are still at the preclinical stage, and comprehensive pharmacokinetic and toxicity data are largely lacking. The inherently dynamic nature of BamA, including its conformational flexibility, also complicates the experimental characterization of functionally relevant binding states. In addition, it can be challenging to distinguish compounds that directly inhibit BamA from those that cause nonspecific disruption of the OM. Finally, studies on resistance evolution are still limited and often do not adequately reflect clinically relevant environments. In particular, the fitness costs measured for BamA resistance mutations under laboratory conditions may not fully predict their behavior *in vivo*, where host-associated selection and compensatory adaptation could modify the fitness and persistence of resistant populations. Longitudinal *in vivo* studies, experimental evolution in host-relevant models, and genomic analysis of resistant isolates are therefore needed to determine whether the fitness constraints observed *in vitro* persist during infection and to define the true evolutionary barrier to BamA resistance.^171^

Current translational gaps extend beyond target validation and antibacterial potency to include exposure, formulation, administration, scalable manufacturing, and long-term safety assessment, particularly for complex macrocyclic peptide scaffolds.

Addressing these gaps will require integrated approaches combining structural biology, microbiology, medicinal chemistry, and translational pharmacology.

To overcome these challenges, future design strategies should emphasize achieving an optimal balance between polarity and lipophilicity to support both solubility and membrane permeability. The use of macrocycles or other conformationally constrained scaffolds can further enhance binding efficiency and target specificity. Whenever possible, prioritizing extracellular BamA regions as binding sites may help bypass the need for full OM traversal. In parallel, incorporating features that promote uptake while reducing susceptibility to efflux will be essential to improve intracellular accumulation and overall antibacterial efficacy.^34,172^

Overall, the successful development of BamA inhibitors requires a highly integrated medicinal chemistry strategy that simultaneously addresses membrane transport, pharmacokinetics, and target engagement within the unique environment of Gram-negative bacteria.

## 7 Future perspectives

The emergence of BamA inhibitors represents a significant paradigm shift in the development of antibiotics targeting Gram-negative pathogens. By disrupting the BAM, particularly BamA, this strategy directly interferes with OM biogenesis—an essential and highly conserved process for bacterial survival. Accumulating structural, biochemical, and pharmacological evidence strongly supports continued expansion of this approach, alongside growing diversity in inhibitor classes such as darobactins, dynobactins, bamabactins, xenorceptides, and lumen-binding macrocycles.^173^

Future progress in this field will depend on the integration of structural biology, medicinal chemistry, computational modeling, and combination therapy strategies to overcome permeability, efflux, and resistance barriers.

### 7.1 Advancing structural elucidation and co-crystallization studies

High-resolution structural characterization will remain central to the rational development of BamA inhibitors. Recent co-crystal and cryo-EM studies have provided detailed insights into ligand–protein interactions and conformational states of BamA. For instance, dynobactin binding has been shown to target the lateral gate and penetrate deeply into the β-barrel lumen, revealing previously unrecognized druggable regions.^40^ Similarly, darobactins and their optimized analogues (*e.g.*, D9, D22) act as β-strand mimetics that exploit the lateral gate as an extended recognition interface.^114,122^

Moreover, macrocyclic peptides such as PTB1 and PTB2 demonstrate state-dependent binding, highlighting the importance of conformational selectivity in inhibitor design.^29^ Complementary studies have also emphasized the role of lipid-mediated conformational dynamics in regulating BamA function, further expanding the landscape of potential binding hotspots.^172^

Continued efforts in co-crystallization and cryo-EM are expected to uncover transient and previously inaccessible conformations, enabling more precise structure-based drug design.

### 7.2 Optimization of macrocyclic scaffolds

Macrocyclic compounds have emerged as one of the most promising classes of BamA inhibitors due to their ability to engage complex and shallow binding interfaces. These molecules can access luminal or extracellular regions of BamA and stabilize inactive conformations, thereby blocking OMP assembly.^34^

Recent advances in synthetic methodologies have significantly improved the accessibility and diversity of macrocyclic scaffolds, facilitating their optimization for enhanced potency and drug-like properties.^6^ Additionally, macrocyclic peptides offer favorable stability profiles and can be fine-tuned to improve selectivity and resistance profiles.^174^ Emerging technologies, including display platforms and incorporation of non-canonical or fluorinated amino acids, further expand the accessible chemical space for BamA-targeting agents.^175,176^

Ongoing work will likely focus on improving pharmacokinetics, OMP, and metabolic stability while preserving strong and selective BamA binding.

### 7.3 Combination therapies targeting outer membrane biogenesis pathways

Combination therapy is expected to play a pivotal role in maximizing the clinical utility of BamA inhibitors. Given the intrinsic permeability barrier of the Gram-negative OM, agents targeting BamA can synergize with antibiotics that act intracellularly.

For example, BamA-targeting small molecules have demonstrated synergy with rifampicin by enhancing membrane permeability.^177^ Similarly, phenotypic compounds such as licochalcone A and nitazoxanide have been reported to perturb OM homeostasis and sensitize bacteria to other antibiotics. However, their precise molecular targets within the BAM system, including whether BamA or BamD is directly engaged, remain insufficiently validated. These compounds should therefore be considered phenotypic or BamA-associated agents rather than confirmed direct BamA inhibitors.^76,178^ Metabolomic studies further confirm that combinations like polymyxin B and rifampicin exert synergistic effects through coordinated disruption of membrane integrity and intracellular processes.^179^ Additionally, non-traditional approaches such as hyperthermia have been shown to impair BamA function and restore antibiotic susceptibility.^180^ Taken together, rationally designed combination regimens targeting multiple nodes of OM biogenesis and function are likely to enhance antibacterial efficacy and reduce resistance development.

### 7.4 Structure-guided design driven by cryo-EM and molecular dynamics

Recent advances in artificial intelligence (AI), MD, and structure-based drug design are transforming BamA inhibitor development. These approaches enable identification of transient binding pockets, allosteric sites, and dynamic conformational states that are difficult to capture experimentally.

AI-based protein structure prediction tools, combined with enhanced sampling simulations, provide critical insights into BamA flexibility and lateral gate dynamics. Molecular docking and MD simulations further facilitate the rational design of β-strand mimetics, conformational stabilizers, and allosteric inhibitors, accelerating lead optimization and reducing experimental cost and time.

Integration of cryo-EM data with AI-driven models and MD simulations now enables a comprehensive framework for studying BamA as a dynamic drug target.^29,34,144,181–185^ The convergence of computational modeling, AI-guided design, and experimental structural biology will establish a powerful pipeline for next-generation BamA inhibitor discovery.

### 7.5 Expanding medicinal chemistry strategies

Future BamA-targeted drug development will also rely on several interconnected medicinal chemistry principles.^118,174,186,187^ Small-molecule β-strand mimetics may be designed to reproduce key substrate interactions, while allosteric inhibitors targeting the POTRA domains could modulate BamA conformational dynamics. Multi-site inhibitors may provide an additional strategy for reducing the likelihood of resistance emergence, whereas targeting extracellular regions could help bypass some of the barriers associated with OM penetration. Optimization of polarity, lipophilicity, and molecular size will remain important for achieving an appropriate balance between permeability and efflux susceptibility. Conformationally constrained scaffolds, including macrocycles, may further enhance binding efficiency, while incorporation of uptake-enhancing features could improve drug accumulation and target exposure.

These strategies collectively define a rational framework for overcoming the intrinsic constraints of Gram-negative antibiotic discovery.

### 7.6 Concluding perspective

Overall, the future of BamA inhibitor development is strongly supported by converging advances in structural biology, computational chemistry, and combination therapy strategies. The ability to exploit dynamic conformational states, design constrained macrocyclic scaffolds, and synergize with existing antibiotics positions BamA as one of the most promising targets in the fight against AMR.

The consistency of findings across multiple disciplines underscores the robustness of this therapeutic strategy and highlights its potential to fundamentally transform antibiotic discovery for Gram-negative pathogens.

## 8 Conclusion

The identification of BamA as a central and essential component of OM biogenesis represents a significant advancement in the search for novel antibacterial targets against Gram-negative pathogens. Its unique structural features, including the dynamic lateral gate and periplasmic POTRA domains, provide multiple opportunities for therapeutic intervention while remaining mechanistically distinct from traditional antibiotic targets. Recent progress in structural and mechanistic understanding has enabled the rational development of diverse classes of BamA inhibitors, including β-strand mimetic macrocycles, conformational modulators, and small molecules. These compounds have not only validated BamA as a druggable target but have also revealed key structure–activity relationships that guide future optimization. Despite these advances, several challenges remain, particularly in achieving efficient OM penetration, minimizing efflux susceptibility, and overcoming potential resistance mechanisms. Addressing these limitations will require a multidisciplinary approach integrating structural biology, medicinal chemistry, and computational design. Future efforts should focus on the development of multi-site and conformationally adaptive inhibitors, as well as combination therapies that enhance membrane permeability and synergize with BamA inhibition. With continued innovation, BamA-targeting strategies hold strong promise for the development of next-generation antibiotics capable of combating multidrug-resistant Gram-negative infections.

## Author contributions

Conceptualization and supervision: Ahmed A. Al-Karmalawy; data collection, data curation, visualization, methodology, and writing – review & editing: Ahmed A. Al-Karmalawy, Arwa Omar Al Khatib, and Samia S. Hawas. All authors approved the submitted version of the manuscript.

## Conflicts of interest

The authors declared no conflict of interest.

## Abbreviations

ADMET: Absorption, distribution, metabolism, excretion, and toxicity
AI: Artificial intelligence
AMR: Antimicrobial resistance
BAM: β-Barrel assembly machinery
Cryo-EM: Cryo-electron microscopy
ESR: Electron spin resonance
HDX-MS: Hydrogen–deuterium exchange mass spectrometry
LPS: Lipopolysaccharide
MD: Molecular dynamics
OM: Outer membrane
OMP: Outer membrane protein
POTRA: Polypeptide transport-associated
PPI: Protein–protein interaction
RND: Resistance-nodulation-division
SAR: Structure–activity relationship
TPR: Tetratricopeptide repeat

## Data Availability

No primary research results, software, or code have been included, and no new data were generated or analyzed as part of this review.
